# Experience of Visiting an Urban Slum: A Medical Student’s Perspective

**DOI:** 10.31729/jnma.8763

**Published:** 2024-09-30

**Authors:** Prabuddha Bajracharya

**Affiliations:** 1 Patan Academy of Health Sciences, Lagankhel, Lalitpur, Nepal

**Keywords:** *community health services*, *health services accessibility*, *patient-centered care*, *slums*

## Abstract

Urban slums are densely populated areas characterized by inadequate housing, poor sanitation, and limited access to essential services. As part of the MBBS curriculum at Patan Academy of Health Sciences (PAHS), we visited an urban slum in Balkhu to assess the community's health-seeking behaviour, water facilities, and education. This article highlights the significance of such postings in equipping medical students with critical skills, including empathy, teamwork, and leadership, necessary for addressing the socio-economic determinants of health. The experience provided an invaluable opportunity to engage with real-world challenges, fostering a deeper understanding of the healthcare needs of underserved populations. These insights are vital to medical students in shaping a compassionate and socially responsible approach in their practice.

## INTRODUCTION

The Bachelor of Medicine and Bachelor of Surgery (MBBS) program encompasses an extensive and comprehensive curriculum that covers numerous medical subjects, making it an expansive course of study.^1^ At Patan Academy of Health Sciences (PAHS), the integrated Community Health Sciences (CHS) forms 25% of the entire curriculum which includes community postings. The postings include five field rotations to urban slums, Health Posts (HPs), Community Diagnosis (CD), Primary Health Care Centers (PHCCs), and district hospitals. The first posting was to an urban slum.

The United Nations (UN) operationally defines a slum as "one or a group of individuals living under the same roof in an urban area, lacking in one or more of the following five amenities".^2^

1. Durable housing (a permanent structure providing protection from extreme climatic conditions);
2. Sufficient living area (no more than three people sharing a room);
3. Access to improved water (water that is sufficient, affordable, and can be obtained without extreme effort);
4. Access to improved sanitation facilities (a private toilet, or a public one shared with a reasonable number of people); and
5. Secure tenure *(de facto or de jure* secure tenure status and protection against forced eviction).

The CHS department divided 65 students into six groups. We were assigned to survey the Jagaran Tole: an urban slum at the banks of the Bagmati River in Balkhu. This article is about experiences of determining the opportunities and the challenges faced by the population who resided in the slum area of Balkhu regarding their health care seeking behaviour, water facility and education.

## PRE-FIELD

The initial expectation among students regarding the posting was to be placed in a healthcare facility with direct patient interaction. However, to our surprise, we were assigned to an urban slum. The orientation sessions that followed, spanning two days, provided a detailed overview of the objectives, outcomes, and etiquettes for the posting. This brought the realization that medicine is not just about treating ailments and prescribing drugs; it is about understanding the broader context in which health and illness exist.^3^ As William Osler famously said, "A good doctor treats the disease; a great doctor treats the patient who has the disease." It became clear that our placement in the slum was not just about treating illnesses, but about understanding and addressing the complex challenges faced by each individual in that community.

As part of our posting, a survey form was made to assess health-seeking behaviour, water facilities, and education in the community. While preparing the questionnaire, all of our group members studied numerous national surveys on health and education. Through this process, we medical students gained valuable insights into the structure and components of a survey form, including the formulation of relevant questions, the organization of sections, and the importance of clear, unbiased language to ensure accurate data collection.

## IN-FIELD

Our field supervisor joined us and introduced us to Mrs. A, a local Female Community Health Volunteer (FCHV), who then guided us on a transect walk. During the conversations, we learned that FCHVs provide their services to the community without receiving any payment. The transect walk was conducted along the road beside the houses, with major landmarks such as the temple, school, and church noted, and environmental features recorded. This information was later used to sketch a social map of the area. The houses were crowded, and being located beside the Bagmati river, the area was highly susceptible to flooding.^4^ The roads were rough and covered in mud. While there was a waste disposal service provided by the municipality weekly, the handling of waste in the area remained a concern. The residents were eager to improve their living conditions but they felt the lack of government support in overcoming these challenges.

Residents were approached at their homes with a warm greeting, and they responded with similar warmth and hospitality. They did not hesitate to give half an hour for filling the survey form. Not just that, people were in fact really cordial and friendly. Despite being medical students, they treated us with respect as future doctors. Many opened up about the daily struggles and hardships of living in the slums, emphasising the need for improved living conditions and access to healthcare. Each household had a unique story, revealing years of hidden pain behind their smiles.

## HEALTH SEEKING BEHAVIOUR AND EDUCATIONAL STATUS

An interview was conducted with Mrs. A to gain insights into the healthcare-seeking behaviour of the community members. She mentioned that there had been a notable improvement in the health status in recent years. Initial observations led to questions about why individuals did not seek hospital care, even when suffering from multiple ailments, and why essential medications were often not purchased. It became evident that the primary barrier was not a lack of willingness to access quality healthcare but rather financial constraints that prevented people from affording such care. Many residents of the slum live below the poverty line, making it difficult for them to afford medical consultations, medications, or even transportation to healthcare facilities. The economic burden of healthcare forces individuals to delay seeking medical help until conditions become critical, often resulting in worsened health outcomes.

This situation often leads to a reliance on traditional healthcare methods. It includes the use of traditional herbs as well as practices like traditional healers. Traditional healers in Nepal are the Dhami-Jhankri (shamans), Pandit-Lama-Gubhaju-Pujari (priests) and Jyotishi (astrologers).^5^ These traditional healers perform rituals, ceremonies, and provide spiritual guidance to address physical, mental, and spiritual ailments in their communities. While these practices are culturally significant, they may delay access to more effective, evidence-based medical treatments. The widespread prevalence of communicable diseases in the slum can be attributed to the lack of proper sanitary conditions. Poor hygiene practices, inadequate sanitation facilities, and contaminated water sources contribute to the high incidence of infectious diseases.

The local school teacher shared that there is a growing understanding of the value of education, leading to most children attending school. However, economic crises and a lack of interest in studies pose barriers to higher education. We spent time interacting with the children and took some pictures together. The teacher revealed enthusiastically about some of his students who had excelled in Information Technology (IT) and nursing sector. He expressed that these achievements motivate him to put in his best efforts, hoping to make a meaningful difference. The assessment of the urban slum revealed numerous obstacles to educational progress, deeply rooted in both socio-economic and environmental factors. Economic challenges were pervasive, with many families struggling to meet basic needs, leading to children being withdrawn from school to contribute to household income. Societal issues, such as child labour, were prevalent, with children being compelled to work instead of attending school, thereby perpetuating the cycle of poverty and limited educational attainment. These multifaceted challenges underscored the complexity of improving educational outcomes in such underserved areas and highlighted the need for targeted interventions at both the community and policy levels.

At last, an exit program was held in Jagaran tole, where we presented our findings to the locals. Creative mediums, such as artwork, tables, and pie charts, were utilized to present the information clearly and engagingly. The community expressed deep gratitude for our efforts and for sharing our findings. It was a bittersweet moment, saying goodbye after building such strong connections.

## TEAMWORK AND LEADERSHIP

Such postings play a crucial role in providing medical students with fundamental abilities that they need outside of the clinical setting. The tasks were distributed among team members and a determined effort was made to finish them through shared learning and collective knowledge effective communication and teamwork. The use of different tools improved data analysis abilities. Interviewing techniques were improved, with a focus on conveying ideas clearly, listening actively, and adapting communication styles to effectively engage with others. Leadership skills were also developed, as students took on roles in guiding the group toward the successful completion of the report. This experience shows how valuable community postings are for developing the wider range of skills needed for practicing medicine.

## THE WAY FORWARD

This posting was not only an opportunity for learning but also a memorable encounter that will leave a lasting impression. The stories shared by the community members serve as a constant reminder of the underlying purpose behind pursuing medicine: to make a tangible difference in the lives of the needy. This experience provided a necessary break from academic study, revealing the ground realities of the community and fostering the essential attributes required in a medical professional. Such postings are instrumental in shaping medical students understanding of the broader context in which health and illness exist, emphasizing the humanistic aspects of medicine.

## References

[1] Anand A, Bammidi S (2014). Medical education and training: implications for india.. Ann Neurosci..

[2] Nolan LB (2015). Slum Definitions in Urban India: Implications for the Measurement of Health Inequalities.. Popul Dev Rev..

[3] Saha S, Beach MC, Cooper LA (2008). Patient centeredness, cultural competence and healthcare quality.. J Natl Med Assoc..

[4] Gautam S, Basnet R (2017). Study on Sanitary Condition in Slum Area of Balkhu, Kathmandu Metropolitancity, Nepal.. International Journal of Medicine and Biomedical Sciences..

[5] Shankar PR, Paudel R, Giri BR (2006). Healing traditions in Nepal.. J Am Assoc Integr Med..

